# The mTOR Signaling Pathway Activity and Vitamin D Availability Control the Expression of Most Autism Predisposition Genes

**DOI:** 10.3390/ijms20246332

**Published:** 2019-12-15

**Authors:** Ekaterina A. Trifonova, Alexandra I. Klimenko, Zakhar S. Mustafin, Sergey A. Lashin, Alex V. Kochetov

**Affiliations:** 1 Federal Research Center Institute of Cytology and Genetics, Siberian Branch of the Russian Academy of Sciences, Novosibirsk 630090, Russia; klimenko@bionet.nsc.ru (A.I.K.); MustafinZS@bionet.nsc.ru (Z.S.M.); lashin@bionet.nsc.ru (S.A.L.); ak@bionet.nsc.ru (A.V.K.); 2 Department of Natural Sciences, Novosibirsk National Research State University, Novosibirsk 630090, Russia

**Keywords:** genetics, bioinformatics, autism spectrum disorder (ASD), SFARI Gene database, mTOR signaling pathway, FMRP, vitamin D3

## Abstract

Autism spectrum disorder (ASD) has a strong and complex genetic component with an estimate of more than 1000 genes implicated cataloged in SFARI (Simon′s Foundation Autism Research Initiative) gene database. A significant part of both syndromic and idiopathic autism cases can be attributed to disorders caused by the mechanistic target of rapamycin (mTOR)-dependent translation deregulation. We conducted gene-set analyses and revealed that 606 out of 1053 genes (58%) included in the SFARI Gene database and 179 out of 281 genes (64%) included in the first three categories of the database (“high confidence”, “strong candidate”, and “suggestive evidence”) could be attributed to one of the four groups: 1. FMRP (fragile X mental retardation protein) target genes, 2. mTOR signaling network genes, 3. mTOR-modulated genes, 4. vitamin D3 sensitive genes. The additional gene network analysis revealed 43 new genes and 127 new interactions, so in the whole 222 out of 281 (79%) high scored genes from SFARI Gene database were connected with mTOR signaling activity and/or dependent on vitamin D3 availability directly or indirectly. We hypothesized that genetic and/or environment mTOR hyperactivation, including provocation by vitamin D deficiency, might be a common mechanism controlling the expressivity of most autism predisposition genes and even core symptoms of autism.

## 1. Introduction

Autism spectrum disorder (ASD) is a heterogeneous neurodevelopmental disorder with complex genetic, environmental, and epigenetic components. The search for genetic factors underlying ASD has led to the identification of more than one thousand genes cataloged in SFARI (Simon’s Foundation Autism Research Initiative) Gene database that has scored and ranked genes into one of the seven categories [1]. The syndromic category includes monogenic mutations that are associated with a substantial degree of increased risk and consistently linked to additional characteristics not required for an ASD diagnosis. Monogenic syndromes have made a tremendous contribution to the study of molecular mechanisms underlying ASD. Dysregulation of the mammalian or mechanistic target of rapamycin (mTORC1) pathway has been identified in numerous syndromes, such as fragile X syndrome, tuberous sclerosis, PTEN (phosphatase and tensin homolog deleted on chromosome 10) hamartoma tumor syndrome, a set of syndromes named RASopathy (includes type 1 neurofibromatosis and other mutations in the RAS/MAPK pathway genes with a very similar manifestation), Angelman syndrome, Rett syndrome, and Phelan–McDermid syndrome (see reviews [2,3]).

The mechanistic target of rapamycin (mTOR) is a serine/threonine kinase and the key translation regulator, the central component of two multiprotein complexes—mTORC1 and mTORC2—which differ in protein compositions and ranges of substrates [3]. The mTOR signaling pathway processes numerous intra and extracellular signals, including growth factors, nutrients, stress, and infections, and participates in the regulation of the immune response, cell and tumor growth, long-term synaptic plasticity, and memory formation [4,5,6]. mTOR signaling has been found to have a pathogenic role in a myriad of neurological disorders, such as epilepsy, autism, intellectual disability, dementia, traumatic brain injury, brain tumors, and hypoxic-ischemic injury [7].

Given the similarities in neuropsychological symptoms between syndromic and idiopathic forms of ASD, mTORC1 dysregulation has been hypothesized to be a common pathological mechanism. Higher activity of mTOR, ERK, and p70S6 kinase and lower activity of GSK3α and tuberin (TSC2) have been observed in cells from children with non-syndromic autism that suggests an increased Akt/mTOR pathway activity in idiopathic ASD [8]. Blood-based microarray studies comparing individuals affected with ASD and typically developing individuals have been used to identify functionally related sets of genes that were over- and under-expressed among ASD samples. The study has demonstrated diminished interferon-, EGF-, PDGF-, PI3K-AKT-mTOR-, and RAS-MAPK-signaling cascades, and increased ribosomal translation in ASD [9]. Consequently, a significant part of both syndromic and idiopathic autism cases can be attributed to disorders caused by mTOR-dependent translation deregulation.

Although mTORC1 affects global protein synthesis, a subset of mRNAs appears to be exceptionally sensitive to changes in mTOR activity [10]. This subset includes several diverse mRNA species: (1) TOP (terminal oligopyrimidine motif) and TOP-like mRNAs via LARP1, (2) mRNAs with short 5′ UTRs enriched for mitochondrial functions, which require EIF4E but are less EIF4A1-sensitive, (3) long 5′ UTR mRNAs encoding proliferation- and survival-promoting proteins, which are both EIF4E- and EIF4A1-sensitive [11]. The percentages of exceptionally sensitive to mTOR activity mRNAs among the autism predisposition genes have never been evaluated before.

RNA-binding protein FMRP, a negative regulator of translation initiation, is one of the key components of the local translation system. The study based on the largest current ASD sample (*n* = 5305) had found only one significantly ASD-associated gene-set consisting of FMRP-targeting transcripts [12]. But FMRP is the target of S6 kinase that is a member of the mTOR pathway, so FMRP-regulated translation is obligatorily dependent on mTOR [13].

Vitamin D3 hormone has been associated with autism based primarily on a correlation between autism incidences in populations with low levels of vitamin D3 [14]. Vitamin D3 is a fat-soluble substance that is converted to its biologically active form 1,25-dihydroxyvitamin D (calcitriol), a steroid hormone that appears to regulate the expression of approximately 900 different genes, a large number of which impact brain development and function [15,16]. Vitamin D3-induced gene regulation involves epigenetic modifications of chromatin conformation at the target loci, as well as reconfiguration of the higher-order chromosomal organization through VDR-mediated recruitment of various regulatory factors [17]. The role of vitamin D3 as a regulator of brain serotonin synthesis has been proposed to explain how low vitamin D3 hormone levels result in aberrant serotonin synthesis, subsequently leading to abnormal brain development [14]. However, the effect of vitamin D3 on ASD symptoms may be also connected with 1,25-dihydroxyvitamin D ability to stimulate expression of DNA damage-inducible transcript 4 (DDIT4), which is a potent mTOR suppressor [18]. We attempted to quantify the percentages of the mTOR signaling network members, the extremely sensitive to mTOR pathway activity targets, FMRP targets, and vitamin D targets among the genes cataloged in the SFARI Gene database.

## 2. Results

### 2.1. SFARI Gene Database Pathway Analysis

**Figure 1:**
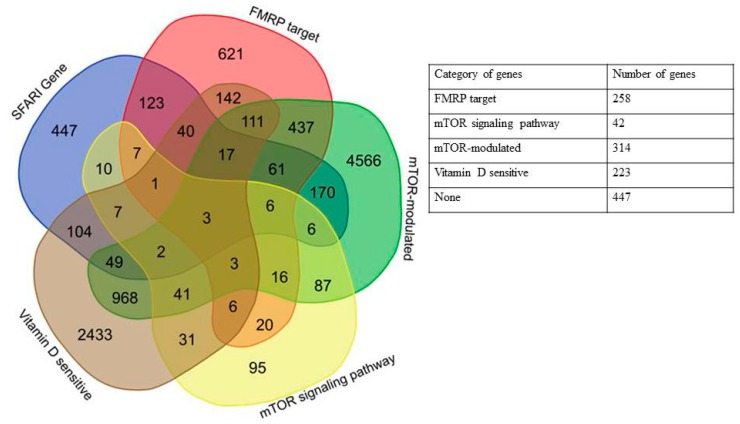
Venn diagram and a generalized table representing the relationships of five categories of genes: SFARI (Simon’s Foundation Autism Research Initiative) Gene database, FMRP (fragile X mental retardation protein) target, mTOR (mechanistic target of rapamycin) signaling network, mTOR-modulated, and vitamin D-sensitive genes.

Here, we showed that 606 out of 1053 genes included in the SFARI Gene database could be attributed to one of the four groups: 1. 258 FMRP target genes, 2. 42 mTOR signaling network genes, 3. 314 mTOR-modulated genes, 4. 223 vitamin D3-sensitive genes, and 447 did not belong to any of the selected categories (see Figure 1). The complete list of SFARI database genes divided into the above categories is given in Supplementary Table S3.

**Figure 2:**
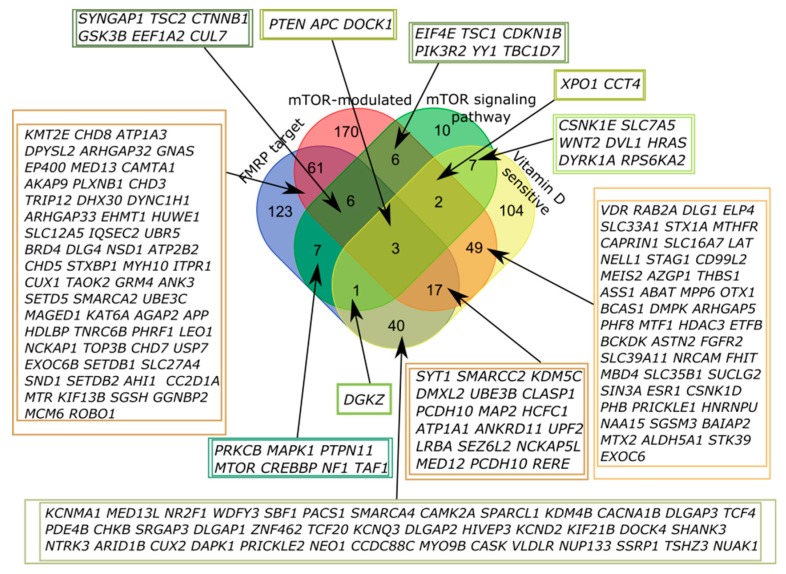
Venn diagram representing the relationship of the four categories related to the mTOR signaling and vitamin D-sensitive genes. All sets of genes were preliminarily intersected with the genes from the SFARI Gene database.

A significant portion of genes that belong to more than one of four categories was of particular interest, with the largest number of intersections observed for FMRP target and mTOR-modulated genes, but it could be attributed to the number of genes in the categories (Figure 2, Table A1). We found that only three out of 1053 genes in the SFARI database fell into all four categories, PTEN (phosphatase and tensin homolog deleted on chromosome 10), APC (adenomatous polyposis coli), and DOCK1 (dedicator of cytokinesis).

Thus, we characterized 58% of autism predisposition genes by dividing them into categories based on their association in the mTOR signaling pathway and vitamin D sensitivity.

**Figure 3:**
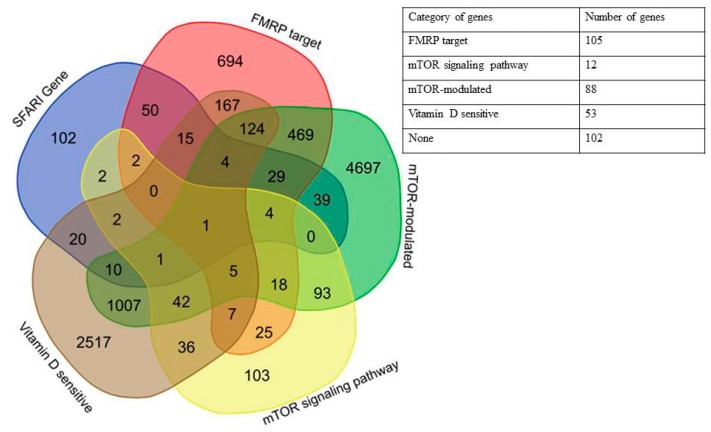
Venn diagram and a generalized table representing the relationships of five categories of genes: SFARI Genes, FMRP target, mTOR signaling network, mTOR-modulated, and vitamin D-sensitive for high scored genes from SFARI Gene database.

It is known that more than half of all scored genes in the SFARI Gene database are placed within the “Minimal Evidence Category” [1]. To check the non-random predominance of the genes associated with mTOR signaling and dependent on vitamin D availability among the autism candidate genes, we additionally analyzed the first three categories of SFARI Gene database (“high confidence”, “strong candidate”, and “suggestive evidence”) containing 281 genes in total. We found that 179 out of 281 genes (64%) could be attributed to one of the four groups (Figure 3), and 102 did not belong to any of the selected categories. The complete list of high scored genes implicated in autism divided into the above categories is given in Supplementary Table S4.

**Figure 4:**
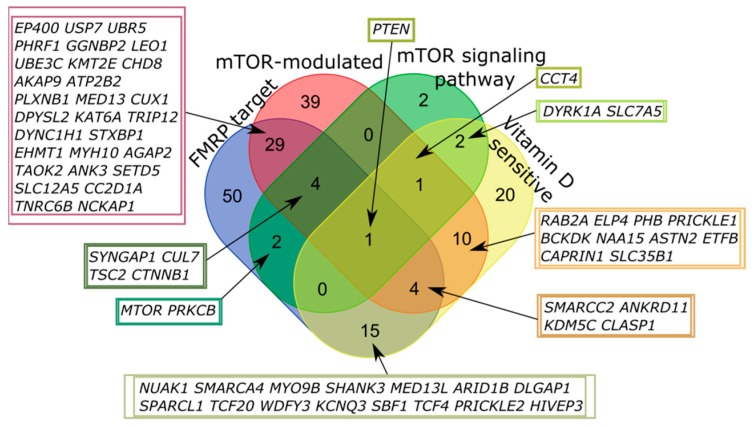
Venn diagram representing the relationship of the four categories related to the mTOR signaling and vitamin D-sensitive genes. All sets of genes were preliminarily intersected with high scored candidates from the SFARI Gene database.

Only one of all high scored genes fell into all four categories, PTEN (phosphatase and tensin homolog deleted on chromosome 10). The largest number of intersections was also observed for FMRP target and mTOR-modulated genes (Figure 4, Table A2).

### 2.2. High Scored Genes from SFARI Gene Database Network Analysis

**Figure 5:**
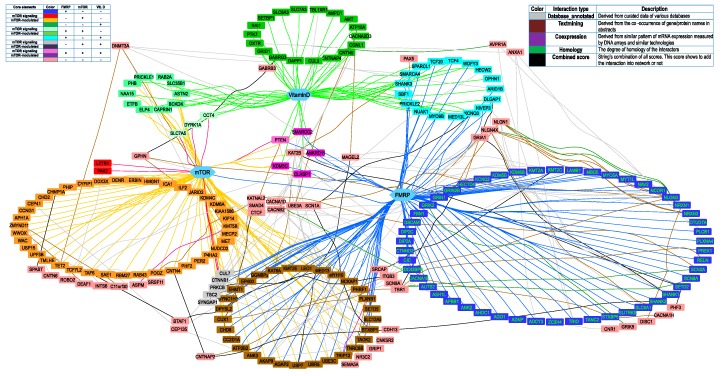
Genes from the first three categories of SFARI Gene database (“high confidence”, “strong candidate”, and “suggestive evidence”) interacting with three core elements (FMRP, mTOR, vitamin D) directly (179 genes) and due to secondary effects (43 genes). All secondarily affected genes are painted pink. The right legend shows the color of the node description. The left legend shows the interaction type colors between 43 secondary genes and genes from the original list.

To obtain more characteristics of the residual 102 out of 281 genes that didn’t interact with mTOR signaling, FMRP, and vitamin D3 directly, we reconstructed a network presenting the interactions between all 281 genes from the first three categories of SFARI Gene database. We were purely interested in the interaction between categorized and residual genes, placed in the first list of 179 and a second list of 102 genes, respectively; the interactions between genes within the same lists were removed. Data on genes/protein interactions were obtained from STRING [19] (see Network construction section) and uploaded into Cytoscape [20,21,22]. First, we visualized 179 genes interacting with three core elements (FMRP, mTOR, vitamin D3), and we found 258 interactions and seven original gene clusters: mTOR-modulated, FMRP-target, vitamin D-sensitive, FMRP target ∩ mTOR-modulated, FMRP target ∩ vitamin D-sensitive, vitamin D-sensitive ∩ mTOR-modulated, and FMRP target ∩ vitamin D-sensitive ∩ mTOR-modulated (Figure 5). These genes were colored by the specter of colors (in the right corner of Figure 5, each cluster of original genes contains 1–3 pluses). Then, we analyzed the second list of 102 genes and finally added to the network 43 new genes and 127 new interactions between them and genes from the first list.

Each of the 43 added genes was connected to genes from the original list by one or more types of interactions (Figure 5, left legend). There are seven types of interactions in the String, with its own score. The interaction was included in the network only if the combined interaction’s score was more than 0.7 (STRING′s “high confidence” cutoff). Only five out of seven interactions types with score >0.7 were found (right legend). Most interactions found were text mining and database annotated. There were only two “experimentally_determined_interactions” (SCN9A – SCN8A and SCN9A – SCN2A), but both of them were also found as “homology” interactions with a higher score, so they were shown on the network as “homology”. There were also 17 interactions with not any separate score of more than 0.7 but with a combined score of more than 0.7. They were marked as “the weakest high confidence” interactions, colored by black.

Thus, we suggested that 222 out of 281 (79%) high scored genes from the SFARI Gene database were related to the mTOR signaling pathway and/or dependent on vitamin D3 availability directly or indirectly (Figure 5).

Interestingly, one of the seven genes (*KATNAL2, SMAD4, CTCF, CACNA1D, CACNB2, UBE3A, SCN1A*) from the second list interacted with three or more original clusters and settled in the middle of the network, notably *UBE3A*, is a well-known gene responsible for the Angelman syndrome that is considered as syndromic form of ASD [23]. Indeed, it has been shown for Angelman syndrome mice that the imbalance between mTORC1 and mTORC2 activity may contribute to synaptic pathology and motor impairment [24].

## 3. Discussion

The prodigious genetic heterogeneity associated with ASD arouses interest to identify common pathways and molecular mechanisms that are responsible for the disorder. Here, we characterized autism predisposition genes by dividing them into four categories directly or indirectly related to the mTOR signaling pathway.

Only three out of 1053 fell into all four categories examined, PTEN (phosphatase and tensin homolog deleted on chromosome 10), APC (adenomatous polyposis coli), and Dock1 (Dock180) (dedicator of cytokinesis). Dock1 (Dock180) protein has been found to play diverse roles in phagocytosis, cell migration, regulation of axon guidance, and dendritic spine morphogenesis [25]. APC is an essential regulator of synaptic adhesion, maturation, and signal transduction networks in forebrain neurons. Strikingly, both *PTEN* and *APC* mutations lead to a similar phenotype, including increased synaptic spine density and altered synaptic function (increased frequency of miniature excitatory synaptic currents, modestly enhanced long-term potentiation) [26,27]. PTEN is an important negative regulator of the AKT/mTOR signaling pathway; neurologically, heterozygous *PTEN* variants are associated with macrocephaly and syndromic autism (PTEN hamartoma tumor syndrome – PHTS) [2]. Deletion of *PTEN* causes 1) a rapid and robust increase in the strength of both long-range and local excitatory inputs and 2) an increase in dendritic length and spine density. Because all these effects were blocked by rapamycin, it was suggested that they occur through the mTORC1 pathway hyperactivation [27]. However, a shift toward higher Akt/mTOR activity has been demonstrated for the general ASD population and not limited to known ASD-associated mTOR pathway genetic mutations [8]. In this study, we reported that 349 out of 1053 SFARI genes (Supplementary Table S3) were directly associated with the mTORC1 pathway, part of them belongs to it, while others depend on it in their translation.

The next category of the autism candidate genes was the targets of FMRP, and 258 out of 1053 SFARI genes (Supplementary Table S3) belonged to it. Recently, gene-set analyses based on the largest ASD sample to date (*n* = 5305) have found only one significantly associated gene-set consisting of FMRP-targeting transcripts (MAGMA: p corr. = 0.014, INRICH: p corr. = 0.031; all competitive *p*-values) [12]. But FMRP itself is a target both of S6 kinase that is regulated by mTORC1 and PP2A phosphatase that is activated in response to stimulation of mGluR receptors [13]. Previously, we conducted in silico experiments and showed that increased activity of the mTOR pathway might result in generating oscillatory (cyclic and quasi-cyclic), chaotic, and even hyperchaotic dynamics of FMRP-dependent postsynaptic protein synthesis. Our results suggested that ASD associated with mTOR pathway hyperactivation might be due to impaired proteome stability associated with complex dynamic regimes of protein synthesis in response to stimulation of mGluR receptors of excitatory synapses [28]. It is highly possible that any mutation in postsynaptic mTOR- or FMRP-targets additionally perturbs proteostasis and worsens autistic phenotype under the condition of mTOR pathway hyperactivation.

Vitamin D hormone is a steroid hormone that appears to regulate the expression of as many as 900 different genes, a large number of which impact brain development and function [15]. Vitamin D deficiency has been linked to many neurological diseases, such as Alzheimer’s disease, Parkinson’s disease, ASD, and multiple sclerosis [14,29]. We found that 223 out of 1053 genes implicated in autism were regulated by 1,25-dihydroxyvitamin D. One of such ASD candidate genes was *TPH2*, coding for tryptophan hydroxylase 2, which is localized in neurons of the raphe nuclei and the enteric nervous system and is the enzyme responsible for producing all of the serotonin in the brain [30]. It was suggested that 1,25-dihydroxyvitamin D activated the transcription of the *TPH2* in the brain at a vitamin D response element (VDRE) and repressed the transcription of *TPH1* in tissues outside the blood-brain barrier at a distinct VDRE. The proposed mechanism explained major characteristics associated with autism and vitamin D deficiency: the low concentrations of serotonin in the brain and its elevated concentrations in tissues outside the blood-brain barrier; the low concentrations of the vitamin D hormone precursor 25-hydroxyvitamin D [25(OH)D3]; the high male prevalence of autism; and the presence of maternal antibodies against fetal brain tissue [14]. At the same time, 1,25-dihydroxyvitamin D upregulates the expression of DNA damage-inducible transcript 4 (*DDIT4*) [18] and phosphatase and tensin homolog deleted on chromosome 10 (*PTEN*) [31], which are potent mTOR suppressors. Thus, vitamin D deficiency has become a major world pandemic [29] and might result in abnormal mTOR activation by itself and increased expressivity of the gene mutations whose transcription is regulated by vitamin D worsened autistic phenotype. The findings that higher concentrations of perinatal/neonatal 25(OH)D3 are associated with a lower risk of ASD additionally support this assumption [32,33,34,35].

One of the genes fallen into three categories examined, *TSC2* (tuberous sclerosis 2), a key regulator of mTOR signaling, causes tuberous sclerosis. The 40%–50% of individuals affected by TSC develop ASD, and one possible explanation for this partial penetrance is an interaction between TSC2 gene mutations and environmental risk factors. It has been shown that maternal immune activation (MIA) and *Tsc2* haploinsufficiency cooperate to disrupt fetal survival and perturb social behavior in adult mice [36]. But mTOR signaling has demonstrated prominent MIA-induced transcriptional dysregulation under targeted network analyses with EIF4E (mTOR-modulated and mTOR-pathway gene in the present study) as one of the most MIA-dysregulated of all ASD-associated genes [37].

The network analysis revealed 43 new genes and 127 new interactions, so on the whole 222 out of 281 (79%) high scored genes from the SFARI Gene database were connected with mTOR signaling activity and/or dependent on vitamin D3 availability directly or indirectly. Thus, one of the 43 genes, UBE3A, interacting with mTOR indirectly and responsible for the Angelman syndrome (AS), also induced hyperactivation of the mTORC1 pathway, resulting in decreased mTORC2 signaling in Purkinje neurons of AS mice. Rapamycin treatment also improves dendritic spine morphology in AS mice, through inhibiting mTORC1 and possibly enhancing mTORC2-mediated regulation of synaptic cytoskeletal elements [24].

It might be hypothesized that genetic and/or environment mTOR hyperactivation, including provocation by vitamin D deficiency, could be a common mechanism controlling penetrance and expressivity of most autism predisposition genes and core symptoms of autism. One of the consequences of the hypothesis was the exceptional importance of promoters and untranslated regions containing the cis-regulatory elements (CRE) necessary for regulation of autism candidate gene expression. Therefore, it is unsurprising that paternally inherited structural variants in CRE were preferentially transmitted to autism-affected offspring and not to their unaffected siblings [38].

The hypothesis was supported by multiple studies demonstrating that pharmacological and natural mTOR-pathway inhibitors improve the behavioral problem, and in the case of vitamin D3 supplementation, even the core symptoms of autism in animal models or even in ASD-affected children [39,40,41,42,43]. The mTOR inhibitor rapamycin has rescued not only the synaptic plasticity but also the behavioral deficits in adult mice with syndromic autism, a heterozygous, inactivating mutation in the TSC2 gene [36]. A particularly promising feature of our hypothesis was a prospective translational effect of ASD classification on the basis of objective genetic information. Given the genetic heterogeneity of ASD and frequent paradoxical response to medication in these patients, it would be extremely helpful to know, for instance, that ASD-related mutation was in a vitamin D3 sensitive gene so that supplementation with high dose vitamin D3 should be proposed first. A less obvious example is the prediction of possible antipsychotic side effects in ASD patients. Second-generation antipsychotic effects in youth have been monitored, and drug-induced parkinsonism rates were as follows: risperidone 16.1% and aripiprazole 27.3% [44], both are recommended for ASD treatment. Network analysis provides evidence of involvement of the mTOR pathway in antipsychotic-induced extrapyramidal symptoms [45], so probably if ASD-related mutation is in gene belonging to the mTOR pathway, the antipsychotic medication should be avoided.

To conclude, our results led to consider ASD as a disorder associated with the deregulation of cap-dependent mTOR-regulated translation. As protein creatures, human beings respond to a variety of environmental stimuli by altering protein biosynthesis. Identifying the major environmental activators of mTOR-regulated translation could help to understand the reasons for the growing rate of ASD and other mTORopathies. Furthermore, a clear connection between ASD and mTOR-mediated translational control also attracts attention to other related mechanisms (e.g., alternative ORFs, eIF2a-mediated control, nonAUG started uORFs, IRES, etc.) that may interfere in some particular cases [46,47,48].

## 4. Materials and Methods

### 4.1. Extracting Genes from Diverse Data Sources

We analyzed the gene sets from the SFARI Gene database [1], KEGG database [49], and from four published studies containing 1. genes that are most reproducibly recognized as FMRP targets [12,50], 2. mTOR-sensitive genes from the NanoCAGE dataset [11], 3. genes included in mTOR signaling network [51], and 4. vitamin D responsive genes and elements [15]. Thus, five sets of genes were identified, and the set theory relations between them were examined in the work:

1. Genes implicated in autism susceptibility (from SFARI Gene database released 01.15.2019 (Supplementary Table S1))–1053 genes;
2. FMRP target genes (1-s2.0-FMRP_tags_842-mmc2.xls [50]–842 genes and Jansen2017.xlsx [12]–1047 genes)–1614 genes;
3. Genes included in the mTOR signaling network (Table S1.xlsx [51]–248 genes and KEGG database–153 genes)–341 genes;
4. mTOR-sensitive genes (mTOR-sensitive 5UTR.xlsx [11])–6543 genes;
5. Vitamin D responsive genes and elements (wang1.xls [15]–902 genes, wang5.xls [15]–3212 genes/loci)–3958 genes/loci.

SFARI Gene 2.0 was used because it includes explicitly defined scoring criteria, continuous updates, and infrastructure to permit community-based involvement [1]. A stringent set of 842 FMRP target transcripts were identified with both antibodies (100%), using both CLIP protocols (100%), different sequencing platforms (100%), and were biologically reproducible (99% were detectable in at least six of seven experiments) [50]. In addition, we included the gene set consisting of FMRP target genes (number of genes 1047), as defined by [12], that partially overlapped with a previous one [50]. A total of 248 genes encoding unique proteins were extracted from the most comprehensive map of the mTOR signaling network [51], and 153 genes were added to the group from the KEGG PATHWAY database that is a collection of manually drawn pathway maps [49].

Transcription start site profiling using nano-cap analysis of gene expression (nanoCAGE) and ribosome-profiling allowed to extract several types of mTOR-modulated mRNAs: (1) TOP (terminal oligopyrimidine motif) and TOP-like mRNAs via LARP1, (2) mRNAs with short 5′ UTRs enriched for mitochondrial functions, which require EIF4E but are less EIF4A1-sensitive, (3) long 5′ UTR mRNAs encoding proliferation- and survival-promoting proteins, which are both EIF4E- and EIF4A1-sensitive [11]. In our analysis, two sets of vitamin D-modulated genes were used: 1. 1,25(OH)_2_D_3_ target genes identified by screening Affymetrix Hu133A Oligonucleotide Microarrays, 2. genes identified by genome screening bearing consensus VDREs or DR3 elements with single-nucleotide substitutions and included elements as being conserved between human and mouse even if they differed in VDRE sequence [15]. All gene sets we analyzed are given in Supplementary Table S2.

### 4.2. Assignment of Genes to Categories and Pathway Analysis

First of all, we were interested in the intersection of the genes implicated in autism susceptibility with various categories of genes related to the mTOR signaling pathway, as well as with the set of vitamin D-sensitive genes. Therefore, the first step was to obtain lists of genes belonging to one of the categories 2–5, which at the same time were among the autism-related genes from the SFARI Gene database. We then analyzed the resulting lists, highlighting the autism genes that appeared in more than one category.

Customized Python scripts were used to perform the list comparison of gene sets and calculate intersections and complements (see Supplementary material). As a key for comparison, a unique identifier “gene symbol” assigned to each of the Homo sapiens genes was used. To take into account possible synonymous gene names, we used the KEGG synonym table (http://rest.kegg.jp/list/hsa) to convert genes symbols into NCBI gene IDs that were used for the consequent comparison. Venn diagrams are widely used in bioinformatics as a tool for the gene-set analysis [52,53]. We used the service http://bioinformatics.psb.ugent.be/webtools/Venn/ for constructing Venn diagrams.

### 4.3. Network Construction

We employed the STRING (Search Tool for the Retrieval of Interacting Genes/Proteins) [19] to identify the relationships among 281 high scored genes from the SFARI Gene database. The reconstruction was made in two steps, based on a unique list of genes and interactions: (1) Visualization of 179 genes. For the three core elements (FMRP, mTOR, vitamin D), we found 179 genes interacting with them and 258 interactions. (2) The imposition of the additional 102 genes that do not interact directly with FMRP, mTOR, and vitamin D. Both lists of (179 + 102) genes were analyzed by STRING to find interactions between them. We used high confidence (0.7) cutoff to filter the interactions. The internal interactions between genes from the second list were also removed, so only the interactions between genes from the first and second lists were added to the network. New genes were clustered in the following way:

(1) If the new gene interacted with one of the core genes, it was settled near this gene.
(2) If the new gene interacted only with one original cluster, it was settled near this cluster.
(3) If the new gene interacted with two original clusters, it was settled between them.
(4) If the new gene interacted with three or more original clusters, it was settled in the middle of a network (seven rose genes).
(5) Finally, the KAT2B gene that interacted with six out of seven original clusters was placed right into the center of a network.

Final network visualization was performed using Cytoscape application [20,21].

## Supplementary Materials

Supplementary materials can be found at https://www.mdpi.com/1422-0067/20/24/6332/s1. Table S1. SFARI-Gene_genes_01-15-2019; Table S2. Complete gene set; Table S3. SFARI genes divided into categories; Table S4-SFARI genes with gene score 1-3 divided into categories.

## Abbreviations

ASD	autism spectrum disorder
SFARI	Simon’s Foundation Autism Research Initiative
mTOR	mechanistic target of rapamycin
FMRP	fragile X mental retardation protein

## Appendix A

**Table A1:** Genes implicated in autism susceptibility that fall into more than one category.

Categories of Genes	Total Number	Elements
FMRP target ∩ vitamin D-sensitive ∩ mTOR signaling pathway ∩ mTOR-modulated	3	PTEN APC DOCK1
FMRP target ∩ mTOR signaling pathway ∩ mTOR-modulated	6	SYNGAP1 TSC2 CTNNB1 GSK3B EEF1A2 CUL7
FMRP target ∩ vitamin D-sensitive ∩ mTOR-modulated	17	SYT1 SMARCC2 KDM5C DMXL2 UBE3B CLASP1 PCDH10 MAP2 HCFC1 ATP1A1 ANKRD11 SEZ6L2 RERE UPF2 LRBA MED12 NCKAP5L PCDH10
FMRP target ∩ vitamin D-sensitive ∩ mTOR- pathway	1	DGKZ
Vitamin D-sensitive ∩ mTOR signaling pathway ∩ mTOR-modulated	2	XPO1 CCT4
FMRP target ∩ mTOR-modulated	61	KMT2E CHD8 ARHGAP32 ATP1A3 DPYSL2 GNAS EP400 CAMTA1 AKAP9 PLXNB1 MED13 CHD3 TRIP12 DHX30 DYNC1H1 ARHGAP33 EHMT1 SLC12A5 IQSEC2 HUWE1 UBR5 BRD4 DLG4 NSD1 ATP2B2 CHD5 STXBP1 MYH10 ITPR1 CUX1 TAOK2 GRM4 ANK3 SETD5 SMARCA2 UBE3C MAGED1 KAT6A AGAP2 APP HDLBP TNRC6B NCKAP1 PHRF1 LEO1 TOP3B CHD7 EXOC6B USP7 SETDB1 SLC27A4 SND1 SETDB2 AHI1 MTR CC2D1A KIF13B SGSH GGNBP2 MCM6 ROBO1
FMRP target ∩ mTOR signaling pathway	7	PRKCB MAPK1 PTPN11 MTOR CREBBP NF1 TAF1
FMRP target ∩ vitamin D-sensitive	40	KCNMA1 MED13L NR2F1 WDFY3 SBF1 PACS1 SMARCA4 CAMK2A SPARCL1 KDM4B CACNA1B DLGAP3 TCF4 PDE4B CHKB SRGAP3 DLGAP1 ZNF462 TCF20 KCNQ3 DLGAP2 HIVEP3 KCND2 KIF21B DOCK4 SHANK3 NTRK3 ARID1B CUX2 DAPK1 PRICKLE2 NEO1 CCDC88C MYO9B CASK VLDLR NUP133 SSRP1 TSHZ3 NUAK1
mTOR-modulated ∩ mTOR-pathway	6	EIF4E TSC1 CDKN1B PIK3R2 YY1 TBC1D7
Vitamin D-sensitive ∩ mTOR-modulated	49	VDR RAB2A DLG1 ELP4 SLC33A1 STX1A MTHFR CAPRIN1 SLC16A7 CD99L2 NELL1 STAG1 LAT MEIS2 AZGP1 MPP6 ASS1 ABAT THBS1 BCAS1 DMPK ARHGAP5 OTX1 PHF8 MTF1 HDAC3 BCKDK ASTN2 SLC39A11 FGFR2 NRCAM FHIT MBD4 ETFB SLC35B1 ESR1 SIN3A SUCLG2 CSNK1D PHB PRICKLE1 MTX2 NAA15 SGSM3 HNRNPU BAIAP2 ALDH5A1 STK39 EXOC6
Vitamin D-sensitive ∩ mTOR signaling pathway	7	CSNK1E SLC7A5 WNT2 DYRK1A DVL1 HRAS RPS6KA2

**Table A2:** High scored genes implicated in autism susceptibility that fall into more than one category.

Categories of Genes	Total Number	Elements
FMRP target ∩ vitamin D-sensitive ∩ mTOR signaling pathway ∩ mTOR-modulated	1	PTEN
FMRP target ∩ mTOR signaling pathway ∩ mTOR-modulated	4	SYNGAP1 CUL7 TSC2 CTNNB1
FMRP target ∩ vitamin D-sensitive ∩ mTOR-modulated	4	SMARCC2 ANKRD11 KDM5C CLASP1
Vitamin D-sensitive ∩ mTOR signaling pathway ∩ mTOR-modulated	1	CCT4
FMRP target ∩ mTOR-modulated	29	EP400 USP7 UBR5 PHRF1 GGNBP2 UBE3C KMT2E CHD8 AKAP9 ATP2B2 PLXNB1 MED13 LEO1 DPYSL2 KAT6A TRIP12 DYNC1H1 STXBP1 EHMT1 MYH10 AGAP2 CUX1 TAOK2 ANK3 SETD5 SLC12A5 CC2D1A TNRC6B NCKAP1
FMRP target ∩ mTOR signaling pathway	2	MTOR PRKCB
FMRP target ∩ vitamin D-sensitive	15	NUAK1 SMARCA4 MYO9B SHANK3 MED13L ARID1B DLGAP1 SPARCL1 TCF20 WDFY3 KCNQ3 SBF1 TCF4 PRICKLE2 HIVEP3
Vitamin D-sensitive ∩ mTOR-modulated	10	RAB2A ELP4 PHB PRICKLE1 BCKDK NAA15 ASTN2 ETFB CAPRIN1 SLC35B1
Vitamin D-sensitive ∩ mTOR signaling pathway	2	DYRK1A SLC7A5

## References

[1] Abrahams B.S., Arking D.E., Campbell D.B., Mefford H.C., Morrow E.M., Weiss L.A., Menashe I., Wadkins T., Banerjee-Basu S., Packer A. (2013). SFARI Gene 2.0: A community-driven knowledgebase for the autism spectrum disorders (ASDs). Mol. Autism.

[2] Winden K.D., Ebrahimi-Fakhari D., Sahin M. (2018). Abnormal mTOR Activation in Autism. Annu. Rev. Neurosci..

[3] Lipton J.O., Sahin M. (2014). The Neurology of mTOR. Neuron.

[4] Lisse T.S., Hewison M. (2011). Vitamin D. Cell Cycle.

[5] Liu Y., Zhang D., Liu X. (2015). mTOR Signaling in T Cell Immunity and Autoimmunity. Int. Rev. Immunol..

[6] Zhou J., Parada L.F. (2012). PTEN signaling in autism spectrum disorders. Curr. Opin. Neurobiol..

[7] Bockaert J., Marin P. (2015). mTOR in Brain Physiology and Pathologies. Physiol. Rev..

[8] Onore C., Yang H., Van de Water J., Ashwood P. (2017). Dynamic Akt/mTOR Signaling in Children with Autism Spectrum Disorder. Front. Pediatr..

[9] Tylee D.S., Hess J.L., Quinn T.P., Barve R., Huang H., Zhang-James Y., Chang J., Stamova B.S., Sharp F.R., Hertz-Picciotto I. (2017). Blood transcriptomic comparison of individuals with and without autism spectrum disorder: A combined-samples mega-analysis. Am. J. Med. Genet. Part B Neuropsychiatr. Genet..

[10] Masvidal L., Hulea L., Furic L., Topisirovic I., Larsson O. (2017). mTOR-sensitive translation: Cleared fog reveals more trees. RNA Biol..

[11] Gandin V., Masvidal L., Hulea L., Gravel S.-P., Cargnello M., McLaughlan S., Cai Y., Balanathan P., Morita M., Rajakumar A. (2016). nanoCAGE reveals 5′ UTR features that define specific modes of translation of functionally related MTOR-sensitive mRNAs. Genome Res..

[12] Jansen A., Dieleman G.C., Smit A.B., Verhage M., Verhulst F.C., Polderman T.J.C., Posthuma D. (2017). Gene-set analysis shows association between FMRP targets and autism spectrum disorder. Eur. J. Hum. Genet..

[13] Narayanan U., Nalavadi V., Nakamoto M., Thomas G., Ceman S., Bassell G.J., Warren S.T. (2008). S6K1 Phosphorylates and Regulates Fragile X Mental Retardation Protein (FMRP) with the Neuronal Protein Synthesis-dependent Mammalian Target of Rapamycin (mTOR) Signaling Cascade. J. Biol. Chem..

[14] Patrick R.P., Ames B.N. (2014). Vitamin D hormone regulates serotonin synthesis. Part 1: Relevance for autism. FASEB J..

[15] Wang T.-T., Tavera-Mendoza L.E., Laperriere D., Libby E., Burton MacLeod N., Nagai Y., Bourdeau V., Konstorum A., Lallemant B., Zhang R. (2005). Large-Scale in Silico and Microarray-Based Identification of Direct 1,25-Dihydroxyvitamin D3 Target Genes. Mol. Endocrinol..

[16] McCann J.C., Ames B.N. (2008). Is there convincing biological or behavioral evidence linking vitamin D deficiency to brain dysfunction?. FASEB J..

[17] Sawatsubashi S., Nishimura K., Mori J., Kouzmenko A., Kato S. (2019). The Function of the Vitamin D Receptor and a Possible Role of Enhancer RNA in Epigenomic Regulation of Target Genes: Implications for Bone Metabolism. J. Bone Metab..

[18] Lisse T.S., Liu T., Irmler M., Beckers J., Chen H., Adams J.S., Hewison M. (2011). Gene targeting by the vitamin D response element binding protein reveals a role for vitamin D in osteoblast mTOR signaling. FASEB J..

[19] Szklarczyk D., Gable A.L., Lyon D., Junge A., Wyder S., Huerta-Cepas J., Simonovic M., Doncheva N.T., Morris J.H., Bork P. (2019). von STRING v11: Protein–protein association networks with increased coverage, supporting functional discovery in genome-wide experimental datasets. Nucleic Acids Res..

[20] Shannon P., Markiel A., Ozier O., Baliga N.S., Wang J.T., Ramage D., Amin N., Schwikowski B., Ideker T. (2003). Cytoscape: A software Environment for integrated models of biomolecular interaction networks. Genome Res..

[21] Su G., Morris J.H., Demchak B., Bader G.D. (2014). Biological Network Exploration with Cytoscape 3. Curr. Protoc. Bioinform..

[22] Smoot M.E., Ono K., Ruscheinski J., Wang P.-L., Ideker T. (2011). Cytoscape 2.8: New features for data integration and network visualization. Bioinformatics.

[23] Peters S.U., Horowitz L., Barbieri-Welge R., Taylor J.L., Hundley R.J. (2012). Longitudinal follow-up of autism spectrum features and sensory behaviors in Angelman syndrome by deletion class. J. Child Psychol. Psychiatry.

[24] Sun J., Liu Y., Moreno S., Baudry M., Bi X. (2015). Imbalanced Mechanistic Target of Rapamycin C1 and C2 Activity in the Cerebellum of Angelman Syndrome Mice Impairs Motor Function. J. Neurosci..

[25] Shi L. (2013). Dock protein family in brain development and neurological disease. Commun. Integr. Biol..

[26] Mohn J.L., Alexander J., Pirone A., Palka C.D., Lee S.-Y., Mebane L., Haydon P.G., Jacob M.H. (2014). Adenomatous polyposis coli protein deletion leads to cognitive and autism-like disabilities. Mol. Psychiatry.

[27] Xiong Q., Oviedo H.V., Trotman L.C., Zador A.M. (2012). PTEN Regulation of Local and Long-Range Connections in Mouse Auditory Cortex. J. Neurosci..

[28] Khlebodarova T.M., Kogai V.V., Trifonova E.A., Likhoshvai V.A. (2018). Dynamic landscape of the local translation at activated synapses. Mol. Psychiatry.

[29] Berridge M.J. (2015). Vitamin D cell signalling in health and disease. Biochem. Biophys. Res. Commun..

[30] Gutknecht L., Kriegebaum C., Waider J., Schmitt A., Lesch K.-P. (2009). Spatio-temporal expression of tryptophan hydroxylase isoforms in murine and human brain: Convergent data from Tph2 knockout mice. Eur. Neuropsychopharmacol..

[31] Shariev A., Painter N., Mason R., Dixon K. PTEN: A novel target for vitamin D in the fight against melanoma. Proceedings of the 16th World Congress on Cancers of the Skin.

[32] Vinkhuyzen A.A.E., Eyles D.W., Burne T.H.J., Blanken L.M.E., Kruithof C.J., Verhulst F., White T., Jaddoe V.W., Tiemeier H., McGrath J.J. (2017). Gestational vitamin D deficiency and autism spectrum disorder. BJPsych Open.

[33] Vinkhuyzen A.A.E., Eyles D.W., Burne T.H.J., Blanken L.M.E., Kruithof C.J., Verhulst F., Jaddoe V.W., Tiemeier H., McGrath J.J. (2018). Gestational vitamin D deficiency and autism-related traits: The Generation R Study. Mol. Psychiatry.

[34] Lee B.K., Eyles D.W., Magnusson C., Newschaffer C.J., McGrath J.J., Kvaskoff D., Ko P., Dalman C., Karlsson H., Gardner R.M. (2019). Developmental vitamin D and autism spectrum disorders: Findings from the Stockholm Youth Cohort. Mol. Psychiatry.

[35] Wu D.-M., Wen X., Han X.-R., Wang S., Wang Y.-J., Shen M., Fan S.-H., Zhuang J., Li M.-Q., Hu B. (2018). Relationship Between Neonatal Vitamin D at Birth and Risk of Autism Spectrum Disorders: The NBSIB Study. J. Bone Min. Res..

[36] Ehninger D., Sano Y., de Vries P.J., Dies K., Franz D., Geschwind D.H., Kaur M., Lee Y.-S., Li W., Lowe J.K. (2012). Gestational immune activation and Tsc2 haploinsufficiency cooperate to disrupt fetal survival and may perturb social behavior in adult mice. Mol. Psychiatry.

[37] Lombardo M.V., Moon H.M., Su J., Palmer T.D., Courchesne E., Pramparo T. (2018). Maternal immune activation dysregulation of the fetal brain transcriptome and relevance to the pathophysiology of autism spectrum disorder. Mol. Psychiatry.

[38] Brandler W.M., Antaki D., Gujral M., Kleiber M.L., Whitney J., Maile M.S., Hong O., Chapman T.R., Tan S., Tandon P. (2018). Paternally inherited cis-regulatory structural variants are associated with autism. Science.

[39] Liu M., Wilk S.A., Wang A., Zhou L., Wang R.-H., Ogawa W., Deng C., Dong L.Q., Liu F. (2010). Resveratrol Inhibits mTOR Signaling by Promoting the Interaction between mTOR and DEPTOR. J. Biol. Chem..

[40] Bhandari R., Kuhad A. (2017). Resveratrol suppresses neuroinflammation in the experimental paradigm of autism spectrum disorders. Neurochem. Int..

[41] Jia F., Wang B., Shan L., Xu Z., Staal W.G., Du L. (2015). Core Symptoms of Autism Improved After Vitamin D Supplementation. Pediatrics.

[42] Jia F., Shan L., Wang B., Li H., Feng J., Xu Z., Saad K. (2019). Fluctuations in clinical symptoms with changes in serum 25(OH) vitamin D levels in autistic children: Three cases report. Nutr. Neurosci..

[43] Mazahery H., Conlon C.A., Beck K.L., Mugridge O., Kruger M.C., Stonehouse W., Camargo C.A., Meyer B.J., Tsang B., Jones B. (2019). A Randomised-Controlled Trial of Vitamin D and Omega-3 Long Chain Polyunsaturated Fatty Acids in the Treatment of Core Symptoms of Autism Spectrum Disorder in Children. J. Autism Dev. Disord..

[44] Carbon M., Kapoor S., Sheridan E., Al-Jadiri A., Azzo S., Sarkaria T., Kane J.M., Saito E., Correll C.U. (2015). Neuromotor Adverse Effects in 342 Youth During 12 Weeks of Naturalistic Treatment with 5 Second-Generation Antipsychotics. J. Am. Acad. Child Adolesc. Psychiatry.

[45] Mas S., Gassó P., Boloc D., Rodriguez N., Mármol F., Sánchez J., Bernardo M., Lafuente A. (2016). Network analysis of gene expression in mice provides new evidence of involvement of the mTOR pathway in antipsychotic-induced extrapyramidal symptoms. Pharm. J..

[46] Kochetov A.V., Allmer J., Klimenko A.I., Zuraev B.S., Matushkin Y.G., Lashin S.A. (2016). AltORFev facilitates the prediction of alternative open reading frames in eukaryotic mRNAs. Bioinformatics.

[47] Kochetov A.V., Prayaga P.D., Volkova O.A., Sankararamakrishnan R. (2013). Hidden coding potential of eukaryotic genomes: NonAUG started ORFs. J. Biomol. Struct. Dyn..

[48] Ventoso I., Kochetov A., Montaner D., Dopazo J., Santoyo J. (2012). Extensive Translatome Remodeling during ER Stress Response in Mammalian Cells. PLoS ONE.

[49] Kanehisa M., Furumichi M., Tanabe M., Sato Y., Morishima K. (2017). KEGG: New perspectives on genomes, pathways, diseases and drugs. Nucleic Acids Res..

[50] Darnell J.C., Van Driesche S.J., Zhang C., Hung K.Y.S., Mele A., Fraser C.E., Stone E.F., Chen C., Fak J.J., Chi S.W. (2011). FMRP Stalls Ribosomal Translocation on mRNAs Linked to Synaptic Function and Autism. Cell.

[51] Caron E., Ghosh S., Matsuoka Y., Ashton-Beaucage D., Therrien M., Lemieux S., Perreault C., Roux P.P., Kitano H. (2010). A comprehensive map of the mTOR signaling network. Mol. Syst. Biol..

[52] Desai M.S., Seekatz A.M., Koropatkin N.M., Kamada N., Hickey C.A., Wolter M., Pudlo N.A., Kitamoto S., Terrapon N., Muller A. (2016). A Dietary Fiber-Deprived Gut Microbiota Degrades the Colonic Mucus Barrier and Enhances Pathogen Susceptibility. Cell.

[53] Sharon G., Cruz N.J., Kang D.-W., Gandal M.J., Wang B., Kim Y.-M., Zink E.M., Casey C.P., Taylor B.C., Lane C.J. (2019). Human Gut Microbiota from Autism Spectrum Disorder Promote Behavioral Symptoms in Mice. Cell.

